# HDGFL2 cryptic proteins report presence of TDP-43 pathology in neurodegenerative diseases

**DOI:** 10.1186/s13024-024-00718-8

**Published:** 2024-03-27

**Authors:** Anna Calliari, Lillian M. Daughrity, Ellen A. Albagli, Paula Castellanos Otero, Mei Yue, Karen Jansen-West, Naeyma N. Islam, Thomas Caulfield, Bailey Rawlinson, Michael DeTure, Casey Cook, Neill R. Graff-Radford, Gregory S. Day, Bradley F. Boeve, David S. Knopman, Ronald C. Petersen, Keith A. Josephs, Björn Oskarsson, Aaron D. Gitler, Dennis W. Dickson, Tania F. Gendron, Mercedes Prudencio, Michael E. Ward, Yong-Jie Zhang, Leonard Petrucelli

**Affiliations:** 1 Department of Neuroscience, Mayo Clinic, Jacksonville, FL USA; 2 Neurobiology of Disease Graduate Program, Mayo Graduate School, Mayo Clinic College of Medicine, Rochester, MN USA; 3 Department of Neurology, Mayo Clinic, Jacksonville, FL USA; 4 Department of Neurology, Mayo Clinic, Rochester, MN USA; 5 Department of Genetics, Stanford University School of Medicine, Stanford, CA USA; 6 National Institute of Neurological Disorders and Stroke, National Institutes of Health, Bethesda, MD USA; 7 Center for Alzheimer’s and Related Dementias, National Institute on Aging and National Institute of Neurological Disorders and Stroke, National Institutes of Health, Bethesda, MD USA

## Abstract

This letter demonstrates the potential of novel cryptic proteins resulting from TAR DNA-binding protein 43 (TDP-43) dysfunction as markers of TDP-43 pathology in neurodegenerative diseases.

**Supplementary Information:**

The online version contains supplementary material available at 10.1186/s13024-024-00718-8.

## To the editor

 Aberrant accumulation of the RNA binding protein TDP-43 in the cytoplasm and its depletion from the nucleus are pathological hallmarks of several neurodegenerative diseases including subsets of frontotemporal lobar degeneration (FTLD-TDP), amyotrophic lateral sclerosis (ALS) and Alzheimer’s disease (AD-TDP). Loss of TDP-43 from the nucleus impairs its ability to repress cryptic exon (CE) inclusion during RNA splicing [1]. Consequently, CEs are anomalously included in critical transcripts such as *STMN2* and *UNC13A* [2–9], which can produce truncated or destabilized RNAs and lead to a loss of their function. Recently, we and others demonstrated that some transcripts with in-frame CEs produce stable CE-containing novel proteins detectable in cerebrospinal fluid (CSF) from patients with FTLD-TDP or ALS [10, 11]. One notable example is a cryptic protein derived from the gene hepatoma-derived growth factor-like protein 2 (HDGFL2-CE), a histone-binding protein expressed throughout the brain. Accordingly, CSF HDGFL2-CE could potentially serve as a sensitive biomarker of TDP-43 pathology, illuminating the contribution of pathological TDP-43 to the clinical variability in TDP-43 proteinopathies [11]. But testing whether CSF HDGFL2-CE is indicative of TDP-43 pathology is hampered by the lack of robust methods to measure pathological TDP-43 in biofluids. To determine if HDGFL2-CE abundance can be used as a readout of the presence of TDP-43 pathology, we probed whether HDGFL2-CE is preferentially expressed in affected neuroanatomical regions with TDP-43 proteinopathy in a cohort of well-characterized post-mortem tissues from FTLD-TDP and AD-TDP cases, and whether HDGFL2-CE abundance associates with pathological TDP-43 burden.

Based on the predicted structure of HDGFL2-CE protein (Fig. S1A), we had generated a previously described rabbit polyclonal HDGFL2-CE antibody (Mayo-LP) [10] that specifically detects HDGFL2-CE but not wild-type HDGFL2 (HDGFL2-WT) proteins in lysates from TDP-43-depleted human induced pluripotent stem cells (iPSC) (Fig. S1B). Using a commercial C-terminal HDGFL2-WT antibody as the capture antibody, and our Mayo-LP HDGFL2-CE antibody as the detection antibody, we then developed a Meso Scale Discovery (MSD) immunoassay that dose-dependently detected endogenous HDGFL2-CE protein in 500‒8000 ng of total protein lysate from TDP-43-depleted iPSC-derived neurons [10]. We have since further optimized the assay by biotinylating the capture antibody, using streptavidin MSD plates, and testing different diluents (Fig. S1C−E). When using MSD Diluent 35, our modified assay detected HDGFL2-CE but not HDGFL2-WT in 16 ng of total protein in lysates from HEK293T cells overexpressing these proteins (Fig. S1D). To determine whether our assay is sufficiently sensitive to detect endogenous HDGFL2-CE, we used lysates from control and TDP-43-depleted iPSCs. Compared to Diluent 35, Diluent 100 provided a better signal to noise ratio detecting endogenous HDGFL2-CE in as little as 125 ng of total protein from TDP-43-depleted iPSC lysates (Fig. S1E).

**Fig. 1:**
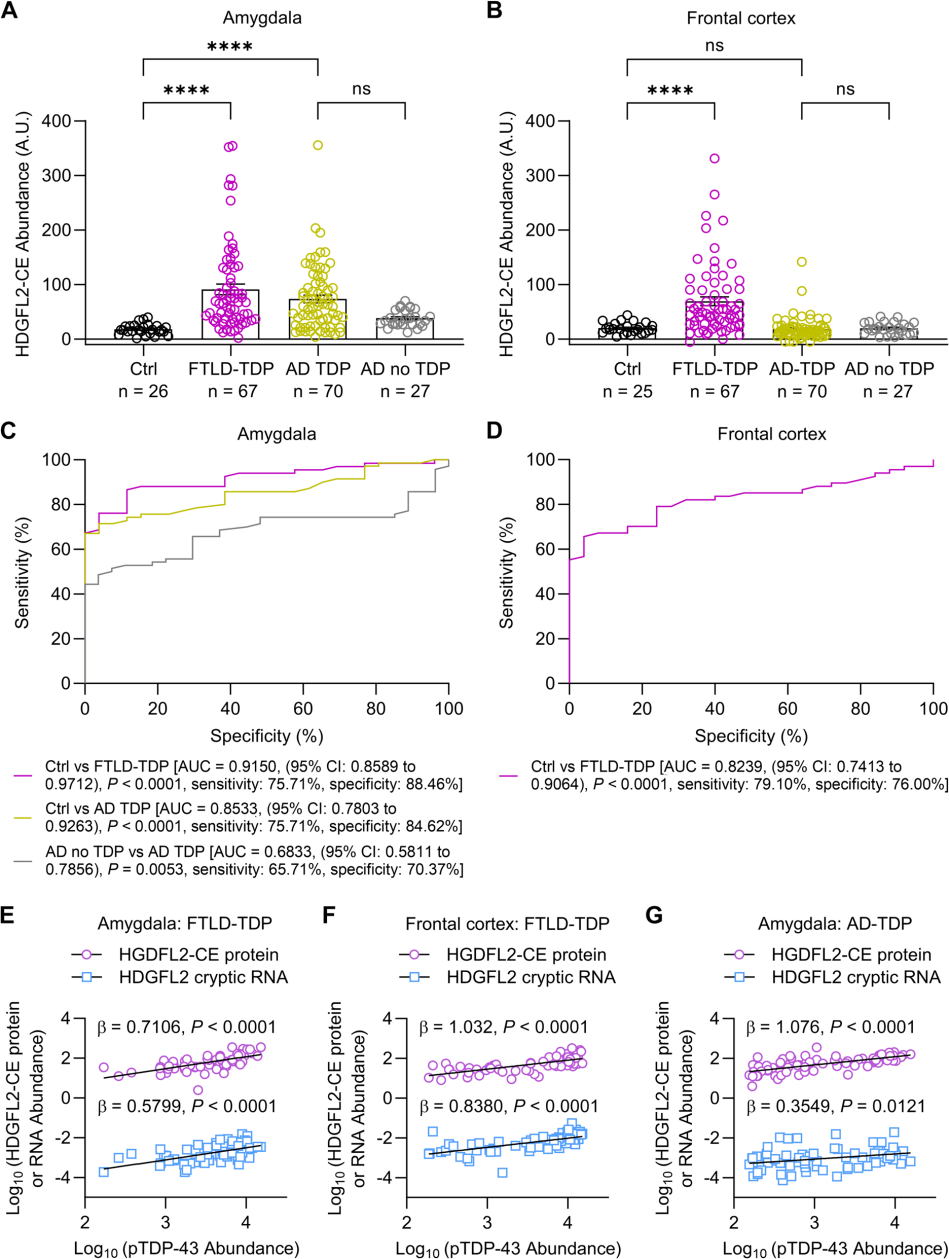
HDGFL2-CE proteins are increased in brain regions with TDP-43 pathology in FTLD-TDP and AD-TDP, and distinguish these TDP-43 proteinopathies from non-TDP-43 controls. Immunoassay quantification of HDGFL2-CE proteins in the amygdala (**A**) and frontal cortex (**B**) of cognitively normal controls (Ctrl, *n *= 27, *n *= 26 amygdala and *n *= 25 frontal cortex), FTLD-TDP (*n *= 67), AD-TDP (*n *= 70) and AD no TDP (*n *= 27). Data are presented as mean ± s.e.m. * *P *< 0.05 and **** *P *< 0.0001, ns: not significant. (**C**, **D**) Area under the receiver operating characteristic curves (AUC) showing the discriminatory capability of HDGFL2-CE in the amygdala or frontal cortex to distinguish FTLD-TDP from Ctrl (pink), AD-TDP from Ctrl (gold), and AD-TDP from AD no TDP (black). AUC values are shown. (**E**–**G**) Scatterplots of HDGFL2-CE protein and RNA abundance with pTDP-43 abundance in the amygdala (**E**) and frontal cortex (**F**) of FTLD-TDP patients, as well as in the amygdala of AD-TDP patients (**G**). Regression coefficients (β) and *P* values from linear regression analysis of pTDP-43 with HDGFL2-CE protein and RNA adjusting for age and sex are shown

 Next, we tested if our optimized assay could detect HDGFL2-CE in brain regions of FTLD-TDP (amygdala and frontal cortex) and AD-TDP (amygdala) characterized by TDP-43 pathology [7]. Compared to cognitively normal controls (controls), HDGFL2-CE was significantly increased in the amygdala of FTLD-TDP and AD-TDP cases in unadjusted analysis and when adjusting for age at death and sex (Fig. 1A, Table S1). In contrast, frontal cortex HDGFL2-CE was significantly increased only in FTLD-TDP cases when compared to controls (Fig. 1B, Table S1). When comparing AD cases without TDP-43 pathology (AD no TDP) to AD-TDP cases, HDGFL2-CE was significantly increased in the amygdala, but not the frontal cortex, in AD-TDP (Fig. 1A, Table S1) – an expected result given the paucity of TDP-43 pathology in the frontal cortex of AD-TDP cases.

The presence of HDGFL2-CE differentiated individuals with and without TDP-43 pathology in the amygdala: HDGFL2-CE distinguished between controls and individuals with TDP-43 pathology with an area under the receiver operating characteristic curve (AUC) of 0.85 and 0.92 for AD-TDP and FTLD-TDP, respectively, indicating good to excellent discriminatory ability (Fig. 1C). When assessing whether amygdala HDGFL2-CE protein distinguishes AD no TDP from AD-TDP, we found moderate discriminatory ability (AUC of 0.68, Fig. 1C). In the frontal cortex, HDGFL2-CE differentiated controls and FTLD-TDP with an AUC of 0.82, indicating good discriminatory ability (Fig. 1D).

Finally, phosphorylated TDP-43 (pTDP-43) burden in the amygdala and frontal cortex significantly associated with HDGFL2-CE protein and *HDGFL2*-CE RNA abundance in FTLD-TDP in both unadjusted analysis and in analysis adjusting for age at death, sex, and RNA integrity number (RIN), the latter for analysis of *HDGFL2*-CE RNA only (Fig. 1E, F, Table S2). In the amygdala of AD-TDP cases, pTDP-43 burden also associated with HDGFL2-CE protein and RNA in unadjusted and adjusted analyses (Fig. 1G, Table S2). However, estimated β coefficients where higher for HDGFL2-CE protein than *HDGFL2*-CE RNA indicating that HDGFL2-CE protein serves as a more accurate indicator of TDP-43 dysfunction.

Retention of CEs in mRNAs owing to TDP-43 dysfunction is well-documented in FTLD-TDP, ALS and AD-TDP [2–8], but the identification that certain in-frame CEs generate stable cryptic proteins is new [8, 10, 11]. Here, we explored the recently identified HDGFL2-CE protein. By developing a sensitive and specific immunoassay to detect HDGFL2-CE proteins, we observed that HDGFL2-CE is significantly increased in brain regions with TDP-43 pathology in FTLD-TDP and AD-TDP, associates with pTDP-43 burden, and can distinguish individuals with TDP-43 pathology from those without. In line with our findings, Irwin et al. used their HDGFL2-CE antibody to perform immunofluorescent staining of motor cortex and hippocampus tissues from patients with ALS-FTD demonstrating that HDGFL2-CE proteins accumulate in cells exhibiting pTDP-43 pathology [11]. They additionally found that, compared to controls, CSF HDGFL2-CE was statistically significantly higher in individuals likely to have TDP-43 pathology, namely presymptomatic or symptomatic C9orf72 repeat expansion carriers and patients with sporadic ALS [11].

Collectively, these findings show that the presence of HDGFL2-CE in the brain is a sensitive reporter of TDP-43 pathology in neurodegenerative diseases. These findings empower CSF HDGFL2-CE as a surrogate marker of TDP-43 pathology and dysfunction, which in turn would inform the selection of ideal participants for clinical trials of potential TDP-43-based therapeutics, and potentially enable precision medicine strategies for pathological subtypes of FTLD and AD.

### Supplementary Information

**Supplementary Material 1.**

## Data Availability

All data generated or analyzed during this study are included in this published article and its supplementary information files, or available from the corresponding authors upon reasonable request.
