# Correction: HDGFL2 cryptic proteins report presence of TDP-43 pathology in neurodegenerative diseases

**DOI:** 10.1186/s13024-024-00744-6

**Published:** 2024-07-27

**Authors:** Anna Calliari, Lillian M. Daughrity, Ellen A. Albagli, Paula Castellanos Otero, Mei Yue, Karen Jansen-West, Naeyma N. Islam, Thomas Caulfield, Bailey Rawlinson, Michael DeTure, Casey Cook, Neill R. Graff-Radford, Gregory S. Day, Bradley F. Boeve, David S. Knopman, Ronald C. Petersen, Keith A. Josephs, Björn Oskarsson, Aaron D. Gitler, Dennis W. Dickson, Tania F. Gendron, Mercedes Prudencio, Michael E. Ward, Yong-Jie Zhang, Leonard Petrucelli

**Affiliations:** 1 Department of Neuroscience, Mayo Clinic, Jacksonville, FL USA; 2 Neurobiology of Disease Graduate Program, Mayo Graduate School, Mayo Clinic College of Medicine, Rochester, MN USA; 3 Department of Neurology, Mayo Clinic, Jacksonville, FL USA; 4 Department of Neurology, Mayo Clinic, Rochester, MN USA; 5 Department of Genetics, Stanford University School of Medicine, Stanford, CA USA; 6 National Institute of Neurological Disorders and Stroke, National Institutes of Health, Bethesda, MD USA; 7 Center for Alzheimer’s and Related Dementias, National Institute on Aging and National Institute of Neurological Disorders and Stroke, National Institutes of Health, Bethesda, MD USA

**Correction: Molecular Neurodegeneration (2024) 19:29**

10.1186/s13024-024-00718-8

The original article contains an error in Figure 1A.

**Figure 1:**
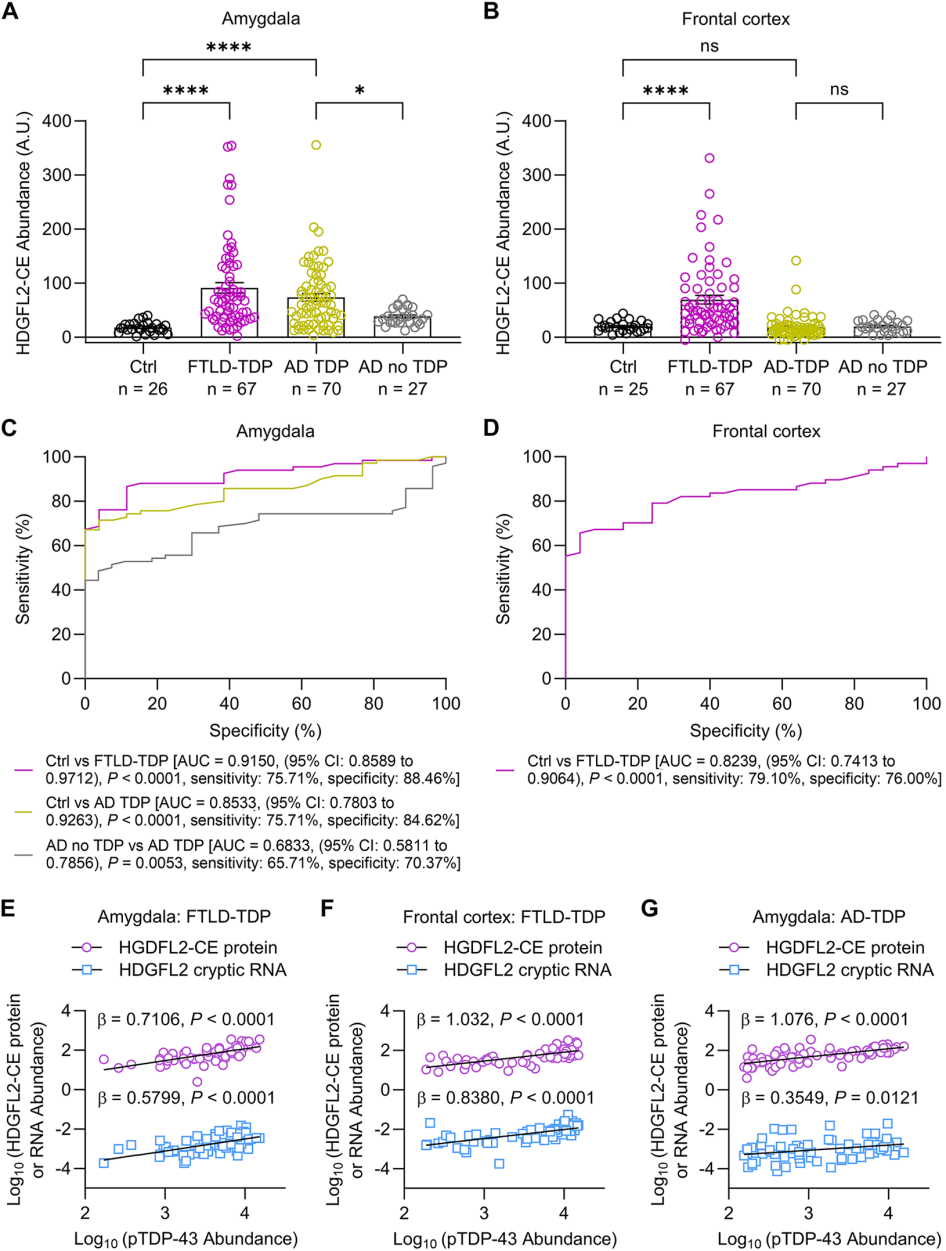

The corrected figure amends the statistical significance annotation of ‘ns’ to ‘*’ and can be viewed ahead.

