# Supplementary material for: HDGFL2 cryptic proteins report presence of TDP-43 pathology in neurodegenerative diseases
Source: Mol Neurodegener. 2024 Mar 27;19:29. doi: 10.1186/s13024-024-00718-8 (PMC10967196; doi:10.1186/s13024-024-00718-8)
Supplement: Supplementary file 1 — Supplementary Material 1. [file 13024_2024_718_MOESM1_ESM.pdf]

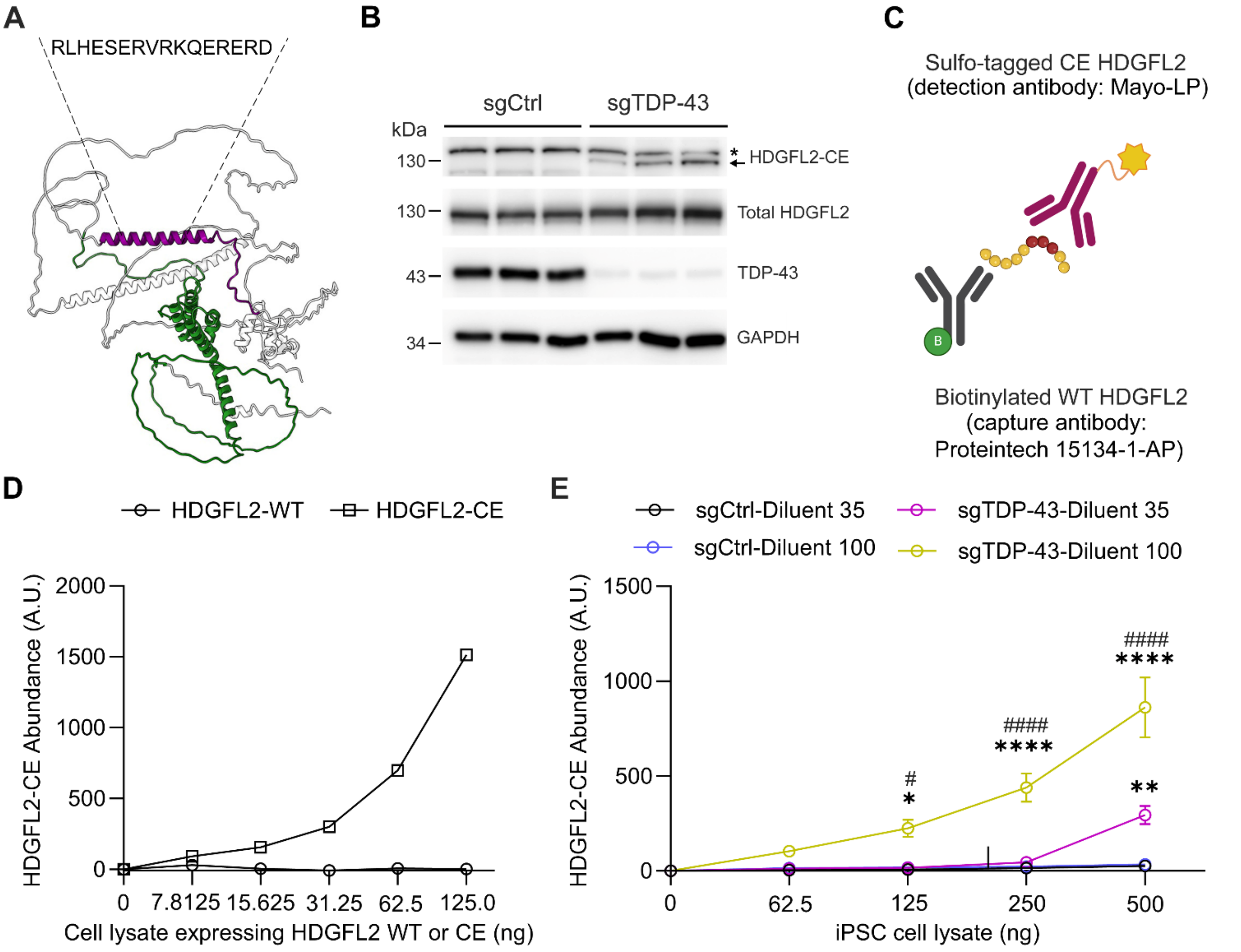

Figure S1. Development of an assay to detect HDGFL2-CE proteins. (**A**) The 3D structure of the HDGFL2-CE predicted by Alphafold is shown. The peptides encoded by the cryptic exon, which localize to an α-helix motif, are labeled in purple and were used to generate the HDGFL2-CE antibody (Mayo-LP). The peptide sequence used for generating a commercial HDGFL2-WT antibody (Proteintech 15134-1-AP) is labeled in green. (**B**) Immunoblot analysis of human iPSC lysates with (sgTDP-43) or without (sgCtrl) TDP-43 depletion using the indicated antibodies. GAPDH was used as a loading control. The arrow represents the HDGFL2-CE band, and an asterisk represents a non-specific band. (**C**) A schematic of the immunoassay we developed on the Meso Scale Discovery platform is shown; a biotinylated Proteintech HDGFL2-WT antibody serves as the capture antibody, and our Sulfo-tag conjugated custom HDGFL2-CE antibody (Mayo-LP) is used as the detection antibody. The schematic was created with BioRender.com. (**D**) HDGFL2-CE protein abundance in lysates from HEK293T cells expressing HDGFL2-CE or HDGFL2-WT constructs as measured by immunoassay. (**E**) HDGFL2-CE protein abundance in sgCtrl or sgTDP-43 iPSC lysates as measured by immunoassay. Diluents 35 and 100 were compared to establish optimal assay conditions (n = 2 independent samples). Data are presented as mean ± s.e.m. $^{*,\#}$ $P < 0.05$, $^{****,\#\#\#\#}$ $P < 0.0001$, Two-way ANOVA, Tukey's multiple comparisons test. * Represents *P* values of the comparisons between sgCtrl vs. sgTDP-43, and # represents *P* values of the comparisons between Diluent 35 vs. Diluent 100 in sgTDP-43-treated cells.

Methods

Three-dimensional modeling of HDGL2 cryptic proteins

The three-dimensional (3D) structure of the HDGFL2 cryptic protein was modeled using AlphaFold 2, which predicts protein folding with high accuracy by leveraging deep learning and extensive sequence alignments [12]. The resulting model provided a structural basis for subsequent analyses. For visualization and detailed examination, the model was processed in ChimeraX, enabling the interactive analysis of molecular structures [13]. Residues of interest were highlighted to visualize key structural elements pertinent to the protein's function. Molecular modeling techniques and tool development have been widely implemented [14].

Antibody generation

The Mayo-LP HDGFL2-CE antibody was generated by Labcorp by immunizing rabbits with a peptide encompassing the 16-residue cryptic epitope (RLHESERVRKQERERD shown in Figure S1A). Pre- and post-immunization sera were collected to confirm specificity and sensitivity prior to affinity purification.

Preparation of cell lysates

Lysates were obtained from HEK293T cells transfected with HDGFL2-WT or HDGFL2-CE constructs. Forty-eight hours post-transfection, cells were harvested and spun at 250 × g for 5 min at 4°C. Pellets were lysed in Co-IP buffer (50 mM Tris–HCl, pH 7.4, 300 mM NaCl, 1% Triton X-100, 5 mM EDTA) with protease and phosphatase inhibitors and sonicated on ice. Supernatant was obtained from high-speed centrifugation (16,000 × g for 20 min) and evaluated using the bicinchoninic acid (BCA) assay (ThermoFisher) to determine protein concentration. Lysates were obtained by transducing the iPSC WTC11 line, harboring stable TO-NGN2 and dCas9-BFP-KRAB cassettes [10], with lentivirus expressing dual guide sgCtrl-sgTDP-43 or sgCtrl-sgCtrl for 24 hours. Following puromycin selection (1 μg/ml, P8833-100MG, Sigma-Aldrich), cells were harvested, lysates were prepared, and protein concentration was determined as described above.

Western Blot

Western blot analysis was conducted by loading 15 μg of total protein from sgCtrl and sgTDP-43 cell lysates into wells of 10% Tris-glycine gels (Novex). The transfer was performed using PVDF membranes (Millipore) pre-soaked with methanol (Pharmco). The membranes were then blocked with 5% nonfat dry milk in TBS with 0.1% Triton X (TBST) for 1 hour followed by overnight incubation with primary antibodies: HDGFL2-CE (1:500, Mayo-LP, AP5820), HDGFL2-WT (1:2000, Proteintech, 15134-1-AP), TDP-43 (1:1000, Proteintech, 12892-1-AP), or GAPDH (1:5000, Meridian Life Science, H86504M). The next day, blots were washed with TBST and incubated with donkey anti-rabbit or anti-mouse horseradish peroxidase-conjugated IgG antibodies (1:5000) for 1 hour. Protein expression was visualized using enhanced chemiluminescence treatment, and images were captured using the ImageQuant 800 (Amersham).

Postmortem brain samples

Postmortem brain tissues were obtained from the Mayo Clinic Florida Brain Bank. Neuropathological diagnoses were conducted for all subjects of the study cohort, as described previously [7]. Amygdala and

frontal cortex tissues were provided from: 27 cognitively normal controls (26 amygdala, 25 frontal cortex) with minimal Braak stage (median: II, range 0-IV) and Thal phase (median: 0, range 0-3), 27 AD cases with no TDP-43 pathology, 70 AD cases with TDP-43 pathology, and 67 FTLD-TDP cases. Note, the presence of pTDP-43 pathology was neuropathologically and immunoassay confirmed [7]. Sample selection was performed balancing for sex (% females: controls=37, AD no TDP=41, AD-TDP=67, FTLD-TDP=48), and based on sample and data availability. Age of death was matched as close as possible among the four study groups (Median: controls=79, range 54-95; AD no TDP=78, range 59-89; AD-TDP=84, range 62-101; FTLD-TDP=71, range 45-91). Note all AD cases had similar Braak stage (Median: AD-TDP=VI, range 4-6; AD no TDP=VI, range V-VI) and Thal phase (Median: AD-TDP=5, range 3-5; AD no TDP=5, range 4-5). FTLD-TDP Braak stage (Median: I-II, range 0-V) and Thal phase (Median: I-II, range 0-V) was lower.

Protein extraction from human brain tissues

RIPA-soluble extracts were generated by homogenizing amygdala and frontal cortex tissues in 5 volumes (w/v) of ice-cold RIPA buffer (25 mM Tris-HCl pH 7.5, 150 mM NaCl, 1% Nonidet P-40, 1% sodium deoxycholate, 0.1% SDS, with protease and phosphatase inhibitors). Homogenates were then sonicated and centrifuged at 100,000 x g for 30 minutes at 4°C, generating RIPA-soluble fractions. Protein concentration was determined using the BCA assay (ThermoFisher).

HDGFL2-CE immunoassay development

The electrochemiluminescent-based Meso-Scale Discovery (MSD) sandwich assay was developed using antibodies against HDGFL2-WT (capture) and HDGFL2-CE (detection) (Figure S1B). Wells of MSD GOLD Streptavidin plates were coated overnight with the biotinylated capture antibody HDGFL2-WT (4 μg/mL, Proteintech, 12892-1-AP). The following day, plates were washed with MSD wash buffer and blocked for 1 hour using 3% MSD Blocker A in TBS-T (Tris-buffered saline with 0.1% Tween) and shaking. HEK293T cell and iPSC lysates were diluted using MSD Diluent 35 or Diluent 100 and loaded in MSD plates at the indicated amounts shown in Figure S1D and S1E. RIPA-soluble extracts from amygdala and frontal cortex human samples were diluted using MSD Diluent 100, loaded onto MSD plates at 35 μg/well, and shaken for 1.5 hours. Plate-bound samples were incubated with Sulfo-tag conjugated HDGFL2-CE antibody (Mayo-LP; 4 μg/mL) for 1 hour to form immunocomplexes prior to adding MSD GOLD Read Buffer A. For each well, the intensity of emitted light, which is reflective of HDGFL2-CE abundance and presented as arbitrary units (A.U.), was acquired upon electrochemical stimulation of the plate using the Meso Scale Discovery QUICKPLEX SQ120. All samples were tested in duplicate, and the person performing the assays did so in a blinded fashion.

***HDGFL2*-CE RNA quantification**

HDGFL2-CE RNA burden was quantified using SYBR GreenER qPCR SuperMix (Invitrogen) on a QuantStudio™ 7 Flex Real-Time PCR System (Applied Biosystems) from previously available samples [7]. Primer sequences were previously validated [10]: *HDGFL2*-CE_forward: 5'-TCACACCTGAGAAGAAAGCAG, *HDGFL2*-CE_reverse: 5'-TCCTCTCTTCTGTGTCCCTCT. Relative quantification of *HDGFL2*-CE RNA was determined using the ΔΔCt method and normalized to endogenous controls: *GAPDH*_forward: 5′-GTTCGACAGTCAGCCGCATC, *GAPDH*_reverse: 5′-

GGAATTTGCCATGGGTGGA; and *RPLP0*_forward: 5′-TCTACAACCCTGAAGTGCTTGAT, *RPLP0*_reverse: 5′-CAATCTGCAGACAGACACTGG.

Statistical Analysis

All statistical analyses were performed in GraphPad Prism 10. Background responses from MSD diluent alone were subtracted from cell and tissue sample responses. HDGFL2-CE protein, pTDP-43 protein, and *HDGFL2*-CE RNA were analyzed on the base 10 logarithmic scale due to their skewed distribution. Separately for the frontal cortex and the amygdala, linear regression models were used to examine associations of HDGFL2-CE protein with study group: 1) FTLD-TDP or AD-TDP vs cognitively normal controls; and 2) AD-TDP vs. AD no TDP. Unadjusted models were first examined, followed by multivariable models that were adjusted for age at death and sex. Associations of frontal cortex or amygdala pTDP-43 protein levels [7] with HDGFL2-CE protein or RNA levels in FTLD-TDP or AD-TDP were assessed using unadjusted models, followed by multivariable models that were adjusted for age at death and sex for HDGFL2-CE protein or age at death, sex and RIN for *HDGFL2*-CE RNA. β values and 95% confidence intervals (CIs) were calculated using either ‘Control’ or ‘AD no TDP’ as the reference level, as indicated in the tables. Significant P values are based on the number of comparisons performed and are provided in table legends. To evaluate the ability of HDGFL2-CE proteins to discriminate AD-TDP or FTLD-TDP cases from controls, or AD-TDP from AD no TDP, we estimated the area under the receiver operating characteristic curve (AUC) along with 95% confidence intervals, % sensitivity and % specificity (CI). Note an AUC value of 0.5 corresponds to predictive ability equal to that of chance, and an AUC of 1.0 represents perfect predictive ability.

Table S1. HDGFL2-CE proteins are significantly increased in brain regions with TDP-43 pathology in FTLD-TDP and AD-TDP patients.

			Unadjusted analysis			***Adjusting for age at death and sex***		
**Group**	**N**	**Median of HDGFL2-CE MSD signal (minimum, maximum)**	**Regression coefficient (95% CI)**	**P-value**	**P value summary**	**Regression coefficient (95% CI)**	**P-value**	**P value summary**
**Amygdala**								
Control	26	17 (1.5, 40)	0.00 (reference)	NA		0.00 (reference)	NA	
FTLD-TDP	67	67.5 (2.5, 354)	0.6317 (0.4580, 0.8055)	<0.0001	****	0.6120 (0.4290, 0.7951)	<0.0001	****
AD-TDP	70	60.75 (4, 355.5)	0.5532 (0.3805, 0.7259)	<0.0001	****	0.5533 (0.3746, 0.7319)	<0.0001	****
AD no TDP	27	33.5 (12.5, 70)	0.00 (reference)	NA		0.00 (reference)	NA	
AD-TDP	70	60.75 (4, 355.5)	0.1749 (0.02122, 0.3286)	0.0262	*	0.1970 (0.02365, 0.3704)	0.0264	*
**Frontal cortex**								
Control	25	18.5 (4.5, 44)	0.00 (reference)	NA		0.00 (reference)	NA	
FTLD-TDP	67	49.5 (-5, 331)	0.4557 (0.2945, 0.6168)	<0.0001	****	0.4095 (0.2425, 0.5764)	<0.0001	****
AD-TDP	70	13.5 (-4.5, 142)	-0.09103 (-0.2518, 0.06975)	0.2651	ns	-0.04062 (-0.2056, 0.1244)	0.6274	ns
AD no TDP	27	19 (3.5, 41.5)	0.00 (reference)	NA		0.00 (reference)	NA	
AD-TDP	70	13.5 (-4.5, 142)	-0.05642 (-0.2099, 0.09706)	0.4671	ns	0.03406 (-0.1296, 0.1977)	0.6802	ns

CI=confidence interval; regression coefficients, 95% CIs, and *P* values are shown from unadjusted linear regression models or linear regression models adjusted for age and sex. β values are interpreted as the difference in the mean levels of HDGFL2-CE proteins between the indicated groups. *P* values < 0.025 (comparisons with Controls) or < 0.05 (Comparison with AD no TDP) are considered statistically significant after correcting for multiple comparisons.

Table S2. pTDP-43 abundance significantly associates with HDGFL2-CE protein and RNA abundance in brain regions with TDP-43 pathology in FTLD-TDP and AD-TDP patients.

		Unadjusted analysis			*Multivariable adjustments*		
**Group**	**N**	**Regression coefficient (95% CI)**	**P-value**	**P value summary**	**Regression coefficient (95% CI)**	**P-value**	**P value summary**
**FTLD-TDP – Amygdala**	67						
HDGFL2-CE protein		0.7174 (0.5155, 0.9192)	<0.0001	****	0.7106 (0.5076, 0.9136)	<0.0001	****
HDGFL2-CE RNA		0.5647 (0.3691, 0.7603)	<0.0001	***	0.5799 (0.3697, 0.7900)	<0.0001	****
**AD-TDP – Amygdala**	70						
HDGFL2-CE protein		1.080 (0.7895, 1.370)	<0.0001	****	1.076 (0.7760, 1.375)	<0.0001	****
HDGFL2-CE RNA		0.3578 (0.0849, 0.6307)	0.0110	*	0.3549 (0.08059, 0.6292)	0.0121	*
**FTLD-TDP – Frontal cortex**	67						
HDGFL2-CE protein		1.060 (0.7806, 1.340)	<0.0001	****	1.032 (0.7521, 1.312)	<0.0001	****
HDGFL2-CE RNA		0.8645 (0.5908, 1.138)	<0.0001	****	0.8380 (0.5623, 1.114)	<0.0001	****

CI=confidence interval; regression coefficients, 95% CIs, and *P* values are shown for associations of pTDP-43 protein and HDGFL2-CE protein or with *HDGFL2-CE* RNA from unadjusted linear regression models or linear regression models adjusted for age, sex (HDGFL2-CE protein) and for age, sex, RNA integrity number (*HDGFL2-CE* RNA). *P* values < 0.025 are considered statistically significant.
